# Mediation of the relationship between proteinuria and serum phosphate: Insight from the KNOW-CKD study

**DOI:** 10.1371/journal.pone.0235077

**Published:** 2020-06-22

**Authors:** Ji Yong Jung, Han Ro, Jae Hyun Chang, Ae Jin Kim, Hyun Hee Lee, Seung Hyeok Han, Tae-Hyun Yoo, Kyu-Beck Lee, Yeong Hoon Kim, Soo Wan Kim, Sue Kyung Park, Dong-Wan Chae, Kook-Hwan Oh, Curie Ahn, Wookyung Chung

**Affiliations:** 1 Department of Internal Medicine, Gachon University Gil Medical Center, Incheon, Republic of Korea; 2 Gachon University College of Medicine, Incheon, Republic of Korea; 3 Department of Internal Medicine, College of Medicine, Institute of Kidney Disease Research, Yonsei University, Seoul, Republic of Korea; 4 Department of Internal Medicine, Sungkyunkwan University School of Medicine, Kangbuk Samsung Hospital, Seoul, Korea; 5 Department of Internal Medicine, Busan Paik Hospital, College of Medicine, Inje University, Busan, Republic of Korea; 6 Department of Internal Medicine, Chonnam National University Medical School, Gwangju, Republic of Korea; 7 Department of Preventive Medicine, Seoul National University College of Medicine, Seoul, Republic of Korea; 8 Department of Internal Medicine, Seoul National University Bundang Hospital, Seoul, Republic of Korea; 9 Department of Internal Medicine, Seoul National University Hospital, Seoul, Republic of Korea; International University of Health and Welfare, School of Medicine, JAPAN

## Abstract

Proteinuria and hyperphosphatemia are risk factors for cardiovascular disease in patients with chronic kidney disease (CKD). Although the interaction between proteinuria and the serum phosphate level is well established, the mechanistic link between the two, particularly the extent to which this interaction is mediated by phosphate-regulating factors, remains poorly understood. In this study, we examined the association between proteinuria and the serum phosphate level, as well as potential mediators, including circulating fibroblast growth factor (FGF23)/klotho, the 24-h urinary phosphate excretion rate to glomerular filtration rate ratio (EP/GFR), and the 24-h tubular phosphate reabsorption rate to GFR ratio (TRP/GFR). The analyses were performed with data from 1793 patients in whom 24-h urine protein and phosphate, serum phosphate, FGF23, and klotho levels were measured simultaneously, obtained from the KoreaN cohort study for Outcome in patients With Chronic Kidney Disease (KNOW-CKD). Multivariable linear regression and mediation analyses were performed. Total, direct, and indirect effects were also estimated. Patients with high serum phosphate levels were found to be more likely to exhibit greater proteinuria, higher FGF23 levels, and lower klotho levels. The 24-h EP/GFR increased and the 24-h TRP/GFR decreased with increasing proteinuria and CKD progression. Simple mediation analyses showed that 15.4% and 67.9% of the relationship between proteinuria and the serum phosphate level were mediated by the FGF23/klotho ratio and 24-h EP/GFR, respectively. Together, these two factors accounted for 73.1% of the relationship between serum markers. These findings suggest that proteinuria increases the 24-h EP/GFR via the FGF23/klotho axis as a compensatory mechanism for the increased phosphate burden well before the reduction in renal function is first seen.

## Introduction

Patients with chronic kidney disease (CKD) exhibit significantly elevated rates of cardiovascular disease–related morbidity and mortality compared with the general population. Proteinuria and hyperphosphatemia have been identified as important kidney-specific risk factors [1–4], although the mechanistic links between these factors and disease outcomes remain poorly understood.

Under normal conditions, proteins that pass through the glomerular filtration barrier are captured in the proximal tubule and transferred to the systemic circulation; this process causes proteinuria in patients with glomerular damage [5, 6]. Phosphate serves as a buffer to help maintain normal serum levels as renal function decreases. As the glomerular filtration rate (GFR) decreases, the serum phosphate concentration is maintained within a normal range via the increased excretion of phosphate. Most of the phosphate that is filtered through the kidney is regulated by sodium and phosphate cotransporters 2a and 2c (NaPi-2a and NaPi-2c), located in the apical membrane of the renal proximal tubule, which is regulated by parathyroid hormone (PTH) and fibroblast growth factor 23 (FGF23) [7].

A previous study showed that phosphate levels remain high, regardless of the GFR, in children with nephrotic-range proteinuria [8]. Another study suggested that FGF23 modifies albuminuria as part of an off-target effect in patients with CKD and immunoglobulin A (IgA) nephropathy [9]. In addition, klotho, a co-receptor of FGF23, was shown to inhibit excess phosphate by interfering with tubular phosphate reabsorption of NaPi-2a [10, 11]. In CKD, phosphate excretion rises as the GFR falls if the process is not accompanied by an influx of phosphate into the systemic circulation (from intestinal absorption, redistribution, and other circulation factors). At the same time, the tubular reabsorption of phosphate may also fall or remain unchanged [12–15]. Kim *et al*. [16] recently reported that patients with CKD and proteinuria >1.0 g/day exhibited higher serum phosphate levels and reduced FGF23 activity, which may comprise a risk factor for CKD progression. In addition, Di Iorio *et al*. [17] reported a potential important interaction by which phosphate interferes with the antiproteinuric response in patients with very-low-protein diets; however, this observation remains controversial, as other researchers have reported that use of a high-phosphate food additive did not directly cause albuminuria [18].

As physiologically filtered phosphate is mostly reabsorbed, a further increase in reabsorption is not uncharacteristic. Phosphate reabsorption and excretion are reduced progressively as renal function deteriorates in patients with CKD. Thus, potential causes of serum phosphate elevation in patients with CKD and proteinuria need to be examined in detail, beyond the simple increase in reabsorption and/or decrease in excretion of phosphate. In this study, we further analyzed the effect of proteinuria on renal phosphate regulation with consideration of potential mediators.

To determine whether this assumption is clinically relevant, we performed a variety of analyses to identify factors affecting the processing of renal phosphate. These analyses included direct assessment of phosphate influx and its association with proteinuria and the serum phosphate level, as well as assessment of whether the association of proteinuria with the serum phosphate level is influenced by potential mediators.

## Materials and methods

### Study design and participants

Data for this study were taken from the KoreaN cohort study for Outcome in patients With Chronic Kidney Disease (KNOW-CKD; NCT01630486 at http://www.clinicaltrials.gov), a multicenter prospective study of a pre-dialysis cohort of 2238 patients with CKD conducted from 2011 to 2016. More detailed overviews of the design and methods of the KNOW-CKD study have been published previously [19].

Study inclusion was limited to patients for whom clinical data on 24-h urine protein and phosphate levels, as well as serum phosphate, FGF23, and klotho levels, had been collected simultaneously. All patients with missing values were removed from our analysis, resulting in a final total of 1793 patients included in this analysis. This study was conducted in accordance with the Declaration of Helsinki, and the research protocol was approved by the institutional review boards of the Seoul National University Hospital (1104–089–359), Seoul National University Bundang Hospital (B-1106/129-008), Yonsei University Severance Hospital (4-2011-0163), Kangbuk Samsung Medical Center (2011-01-076), Seoul St. Mary’s Hospital (KC11OIMI0441), Gachon University Gil Medical Center (GIRBA2553), Eulji General Hospital (201105–01), Chonnam National University Hospital (CNUH-2011-092), and Pusan Paik Hospital (11–091). Written informed consent was obtained from all subjects.

### Variables

Data on patients’ sociodemographic characteristics, smoking status, medical histories, medication use, and health-related behaviors were collected using a self-administered questionnaire with the assistance of trained staff. Blood samples were collected after fasting for at least 8 h. Random midstream urine samples were collected using a standard protocol and sent to the central laboratory of the KNOW-CKD study (Lab Genomics, Seongnam, Republic of Korea). Serum creatinine levels were measured using traceable isotope dilution mass spectroscopy. Estimated glomerular filtration rates (eGFRs) were calculated using the Chronic Kidney Disease Epidemiology Collaboration equation [20].

### Measurements

Serum intact FGF23 and C-terminal fibroblast growth factor 23 (cFGF23) levels were measured using an enzyme-linked immunosorbent assay (ELISA; Immunopics, Inc., Athens, OH, USA). The serum α-klotho level was measured using a commercially available ELISA kit (Immuno Biological Laboratories Co., Ltd.).

The fractional excretion of phosphate (FEP) was defined as the ratio of phosphate clearance to creatinine clearance: FEP (%) = (urine phosphate × serum creatinine) × 100 / (serum phosphate × urine creatinine). To quantify the possible phosphate excretion and reabsorption at the time of serum sampling for the measurement of phosphate, the 24-h excretion of phosphate (EP) was calculated using the formula: 24 h EP (mg/day) = 24-h urinary phosphate level (mg/day). The 24-h tubular reabsorption rate of phosphate (TRP) was calculated using the formula: 24 h TRP (mg/day) = 14.4 × GFR (mL/min) × serum phosphate (mg/dL)– 24-h EP (mg/day). Those factors, corrected for the GFR, were reported as 24-h EP/GFR and 24-h TRP/GFR, respectively.

The daily protein intake (DPI) was estimated using the 24-h urinary urea nitrogen (UUN) level following Maroni *et al*. [21]: DPI (g/day) = 6.25 × (24-h UUN (g/day) + 0.031 (g/kg/day) × ideal body weight (kg), where the ideal body weight was calculated assuming the optimal body mass index (BMI) of 22.5 kg/m^2^ [22]. The DPI was expressed as the normalized estimated DPI divided by ideal body weight (g/kg/day).

### Statistical analysis

Normally distributed data are expressed as means ± standard deviations, and variables with skewed distributions are expressed as medians (interquartile ranges). Categorical data are expressed as numbers (percentages). Differences between groups were analyzed using Student’s *t* test, the Mann–Whitney *U* test, the chi-squared test, one-way analysis of variance, and the Kruskal–Wallis test, as appropriate. To study the association between proteinuria and the serum phosphate level, we performed stepwise multiple linear regression analyses. Proteinuria is expressed as 24-h urinary protein excretion (UPE) in categories (<30, 30–300, 300–1000, and ≥1000 mg/day). The DPI; high-sensitivity C-reactive protein (hsCRP), FGF23, and klotho levels; and 24-h EP/GFR were natural log–transformed due to skewed distributions.

We performed mediation analyses with the PROCESS macro [version 3; model = 4, simple mediator model (S1A Fig in S1 Data); model = 6, serial two-mediator model (S1B Fig in S1 Data)] and various measures of effect size for indirect, direct, and total effects, along with bootstrapping with 10,000 resamples for confidence interval (CI) calculation using the method described by Hayes [23]. All analyses were performed using IBM SPSS (version 23.0; IBM Corporation, Armonk, NY, USA) and R (version 3.5.1; Vienna, Austria) software. Two-sided *p* values ≤ 0.05 were considered to be significant. To correct for multiple comparisons across mediations, Bonferroni correction or bootstrapping were applied.

## Results

### Baseline characteristics

Data from a total of 1793 patients were analyzed by serum phosphate quartile. The mean age of the patients was 54 years, and 60.2% of the participants were male. Other baseline characteristics are listed in Table 1.

**Table 1:** Baseline characteristics of study subjects according to quartiles of serum phosphate.

Variables	Overall (n = 1793)	Q1 ≤ 3.2 mg/dL (n = 460)	Q2 3.2–3.6 mg/dL (n = 443)	Q3 3.6–4.1 mg/dL (n = 426)	Q4 ≥ 4.1 mg/dL (n = 464)	P
Age, yr	54.0 ± 12.0	53.1 ± 12.4	54.4± 12.1	53.8 ± 11.9	54.5 ± 11.7	0.169
Male, n (%)	1079 (60.2)	350 (76.1%)	309 (69.8%)	223 (52.3%)	197 (42.5%)	< 0.001
BMI, kg/m^2^	24.6 ± 3.4	24.5 ± 3.1	24.7 ± 3.1	24.7 ± 3.8	24.5 ± 3.6	0.879
Smoking, n (%)	842 (47.0)	260 (56.5%)	233 (52.7%)	178 (41.8%)	171 (36.9%)	< 0.001
DM, n (%)	628 (35.0)	116 (25.2%)	129 (29.1%)	158 (37.1%)	225 (48.5%)	< 0.001
HTN, n (%)	1724 (96.2)	442 (96.1%)	423 (95.5%)	405 (95.1%)	454 (97.8%)	0.140
CVD, n (%)	297 (16.6)	74 (16.1%)	79 (17.8%)	75 (17.6%)	69 (14.9%)	0.596
CCI	3.4 ± 2.2	3.1 ± 2.2	3.3 ± 2.2	3.4 ± 2.2	4.0 ± 2.2	< 0.001
GFR, ml/min/1.73m^2^	53.1 ± 30.8	61.9 ± 28.1	58.4 ± 29.0	55.0 ± 31.3	37.6 ± 28.8	< 0.001
24hr UPE, mg/day*	540.0 (168.0–1566.6)	393.5 (133.6–928.4)	436.0 (132.9–1189.0)	567.2 (175.0–1670.0)	991.3 (319.8–2415.6)	< 0.001
RAAS blockers, n (%)	1539 (85.8)	390 (84.8%)	386 (87.1%)	358 (84.0%)	405 (87.3%)	0.397
Diuretics, n (%)	574 (32.0)	98 (21.3%)	121 (27.3%)	137 (32.2%)	218 (47.0%)	< 0.001
CCBs, n (%)	776 (43.3)	173 (37.6%)	178 (40.2%)	172 (40.4%)	253 (54.5%)	< 0.001
β-blockers, n (%)	446 (24.9)	88 (19.1%)	107 (24.2%)	101 (23.7%)	150 (32.3%)	< 0.001
Hg, g/dL	12.8 ± 2.0	13.7 ± 1.9	13.5 ± 1.8	12.7 ± 1.8	11.6 ± 1.7	< 0.001
Albumin, g/dL	4.2 ± 0.4	4.2 ± 0.4	4.2 ± 0.4	4.2 ± 0.4	4.1 ± 0.5	< 0.001
TC, mg/dL	173.7 ± 39.1	171.6 ± 36.0	172.4 ± 41.8	175.2 ± 38.0	175.5 ± 40.5	0.082
hsCRP, mg/L*	0.6 (0.2–1.6)	0.6 (0.2–1.7)	0.6 (0.3–1.7)	0.6 (0.2–1.5)	0.6 (0.2–1.7)	0.620
Ca, mg/dL	9.1 ± 0.5	9.1 ± 0.5	9.2 ± 0.4	9.2 ± 0.5	9.0 ± 0.7	< 0.001
P, mg/dL	3.7 ± 0.7	2.9 ± 0.3	3.5 ± 0.1	3.8 ± 0.1	4.5 ± 0.5	< 0.001
25D, ng/mL	18.1 ± 10.1	19.9 ± 11.2	19.0 ± 9.6	17.7 ± 10.1	15.8 ± 8.8	< 0.001
1,25D, pg/mL	31.6 ± 16.8	34.6 ± 16.7	33.5 ± 16.3	30.6 ± 17.9	27.5 ± 15.3	< 0.001
PTH, pg/mL	75.1 ± 77.7	61.2 ± 59.1	56.5 ± 39.7	66.0 ± 56.7	113.8 ± 114.1	< 0.001
FGF23, RU/mL*	19.7 (1.6–34.7)	12.2 (0.5–28.8)	18.6 (1.1–31.7)	19.9 (3.9–33.4)	26.9 (5.4–48.7)	< 0.001
Klotho, pg/mL*	535.0 (420.0–667.0)	545.5 (441.5–687.0)	559.0 (420.0–689.5)	530.0 (424.0–664.0)	503.0 (392.5–625.5)	0.001
FGF23/Klotho, RU/ng*	33.8 (2.8–73.2)	22.1 (0.9–56.9)	31.6 (2.2–61.5)	34.7 (7.4–70.4)	53.6 (10.2–111.8)	< 0.001
FEP, %	19.6 ± 11.9	17.6 ± 10.1	18.0 ± 10.6	17.7 ± 10.8	24.7 ± 14.2	< 0.001
DPI, g/kg/day*	0.9 (0.8–1.1)	1.0 (0.8–1.2)	1.0 (0.8–1.2)	0.9 (0.8–1.1)	0.9 (0.7–1.1)	< 0.001
24hr EP, mg/day*	570.4 (400.0–744.8)	600.0 (456.6–800.0)	600.0 (400.0–800.0)	552.2 (400.0–743.2)	500.0 (400.0–685.0)	< 0.001
24hr EP/GFR, mg/day/GFR*	12.3 (7.3–20.6)	10.5 (7.0–16.3)	10.9 (6.6–17.1)	11.6 (7.2–19.2)	18.1 (10.0–30.9)	< 0.001
24hr TRP, mg/day*	1689.0 (899.0–2967.8)	1693.2 (1003.4–2730.4)	1999.6 (1143.4–3214.1)	1946.0 (1007.9–3644.8)	1252.5 (544.6–2477.5)	< 0.001
24hr TRP/GFR, mg/day/GFR*	38.7 (29.7–45.7)	30.9 (24.7–36.0)	38.8 (33.3–43.1)	43.7 (35.8–48.1)	45.8 (35.7–53.5)	< 0.001

*Data are expressed as median and interquartile range, and compared using the Kruskal-Wallis test.

BMI: body mass index; DM: diabetes mellitus; HTN: hypertension; CVD: cardiovascular disease; CCI: Charlson comorbidity index, GFR: estimated glomerular filtration rate by CKD-EPI formula; 24hr UPE: 24hr urine protein; RAAS: renin-angiotensin-aldosterone system; CCB: calcium channel blocker; Hg: hemoglobin; TC: total cholesterol; hsCRP: highly sensitive C-reactive protein; Ca: calcium; P: phosphate; 1,25D: 1,25-(OH)_2_-vitamin D3; 25D: 25-(OH)-vitamin D3; PTH: parathyroid hormone; FGF23: fibroblast growth factor23; FEP: fractional excretion of phosphate; DPI: estimated daily protein intake; 24hr EP/GFR: 24hr urinary phosphate excretion rate of phosphate to glomerular filtration rate; 24hr TRP/GFR; 24hr tubular reabsorption of phosphate to glomerular filtration rate

With increasing serum phosphate level, patients exhibited steady increases in the level of FGF23 and the 24-h EP/GFR and 24-h TRP/GFR, combined with decreases in the klotho level and DPI (Table 1).

### Proteinuria, the FGF23/klotho ratio, the 24-h EP/GFR, and the serum phosphate level

Table 2 shows crude and adjusted relationships between 24-h UPE and the serum phosphate level. Coefficients (Coeff.) and standard errors (SEs) are expressed according to increasing 24-h UPE categories (<30, 30–300, 300–1000, and ≥1000 mg/day). The association between proteinuria and the serum phosphate level was maintained well in the adjusted analysis relative to the crude analysis (Coeff. ± SE, 0.153 ± 0.017; *p* < 0.001; Table 2) after controlling for age, sex, smoking, BMI, diabetes mellitus (DM), hypertension (HTN), Charlson comorbidity index (CCI), renin-angiotensin-aldosterone system blocker use, PTH level, DPI, hsCRP level, and eGFR. In model 4, with the addition of the FGF23/klotho ratio and 24-h EP/GFR, the association was no longer significant, as evidenced by a significant decrease in the correlation coefficient (Coeff. ± SE, 0.017 ± 0.022; *p* = 0.444; Table 2).

**Table 2:** Univariate and adjusted associations between proteinuria (exposure) and serum phosphate level (outcome) (n = 1701).

Model	*Coeff*. *± SE*	*t value*	*P*
**Crude**	0.153 ± 0.017	9.260	< 0.001
**Model 1**	0.168 ± 0.016	10.313	< 0.001
**Model 2**	0.114 ± 0.017	6.757	< 0.001
**Model 3**	0.044 ± 0.021	2.077	0.038
**Model 4**	0.017 ± 0.022	0.766	0.444

Model 1: adjustment for age, sex, smoking, and BMI

Model 2: model 1+adjustment for DM, HTN, and CCI

Model 3: model 2+adjustment for RAAS blockers, PTH, DPI, hsCRP and eGFR

Model 4: model 3+adjustment for FGF23/Klotho and 24hr EP/GFR.

Estimate coefficient (Coeff.) and standard Error (SE) are expressed according to increasing 24hr UPE categories (<30, 30–300, 300–1000, ≥1000 mg/day).

DPI, hsCRP, FGF23, Klotho and 24hr EP/GFR have been natural log-transformed due to skewed distribution.

**Fig 1:**
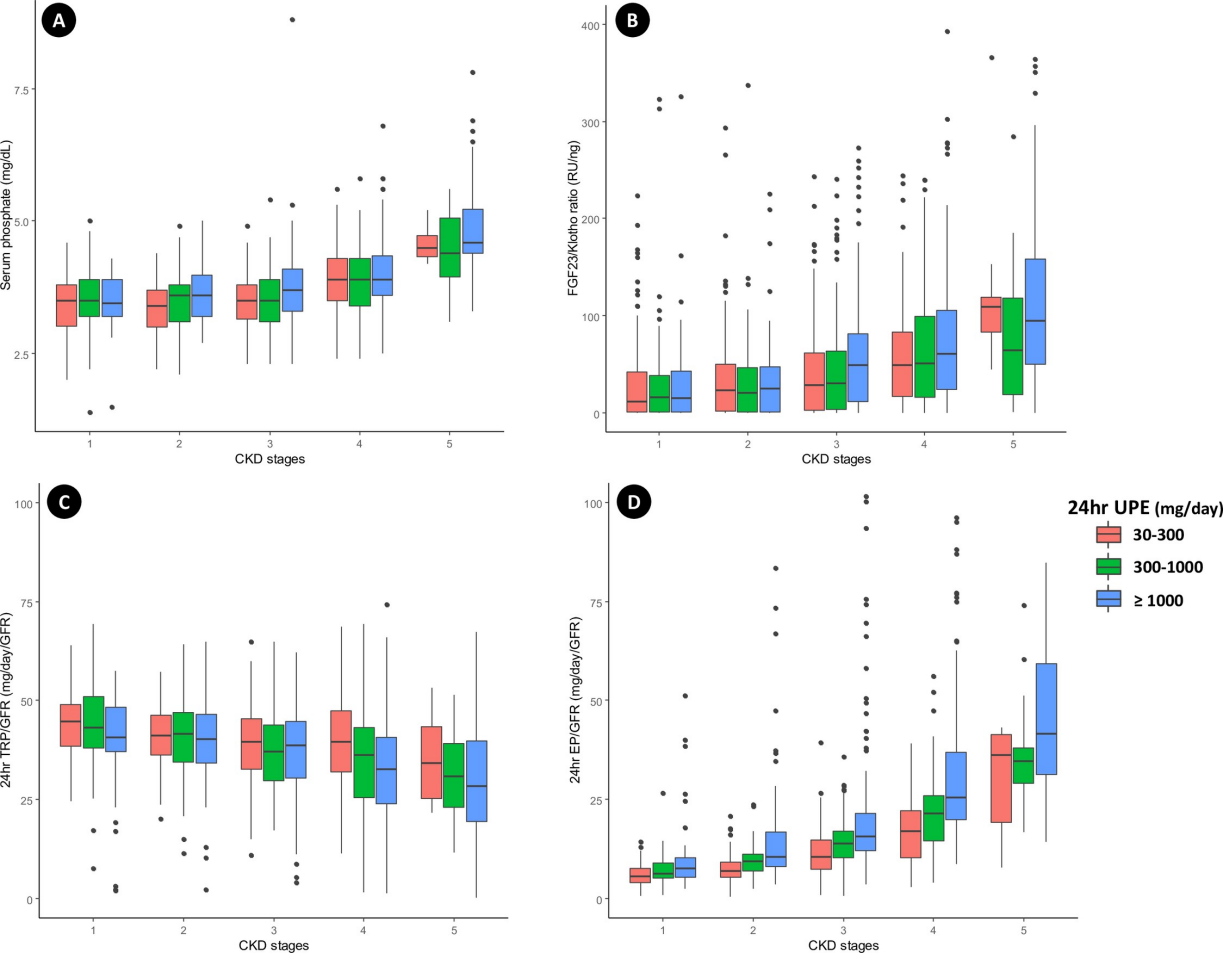
Associations among the serum phosphate level, FGF-23/klotho ratio, 24-h EP/GFR, and 24-h TRP/GFR according to CKD stage and degree of proteinuria. Patients with CKD exhibit higher serum phosphate levels, FGF-23/klotho ratios, and 24-h EP/GFRs and lower 24-h TRP/GFRs with increasing proteinuria. The relationships between the CKD stage and (A) serum phosphate level, (B) FGF-23/klotho ratio, (C) 24-h TRP/GFR, and (D) 24-h EP/GFR in three 24-h UPE groups were plotted. The central rectangles in the boxplots span the interquartile ranges. The segments inside the rectangles show the medians. Whiskers above and below the boxes show the locations of the minimum and maximum values, which are no more than 1.5 times the interquartile ranges distant from the boxes.

**Fig 2:**
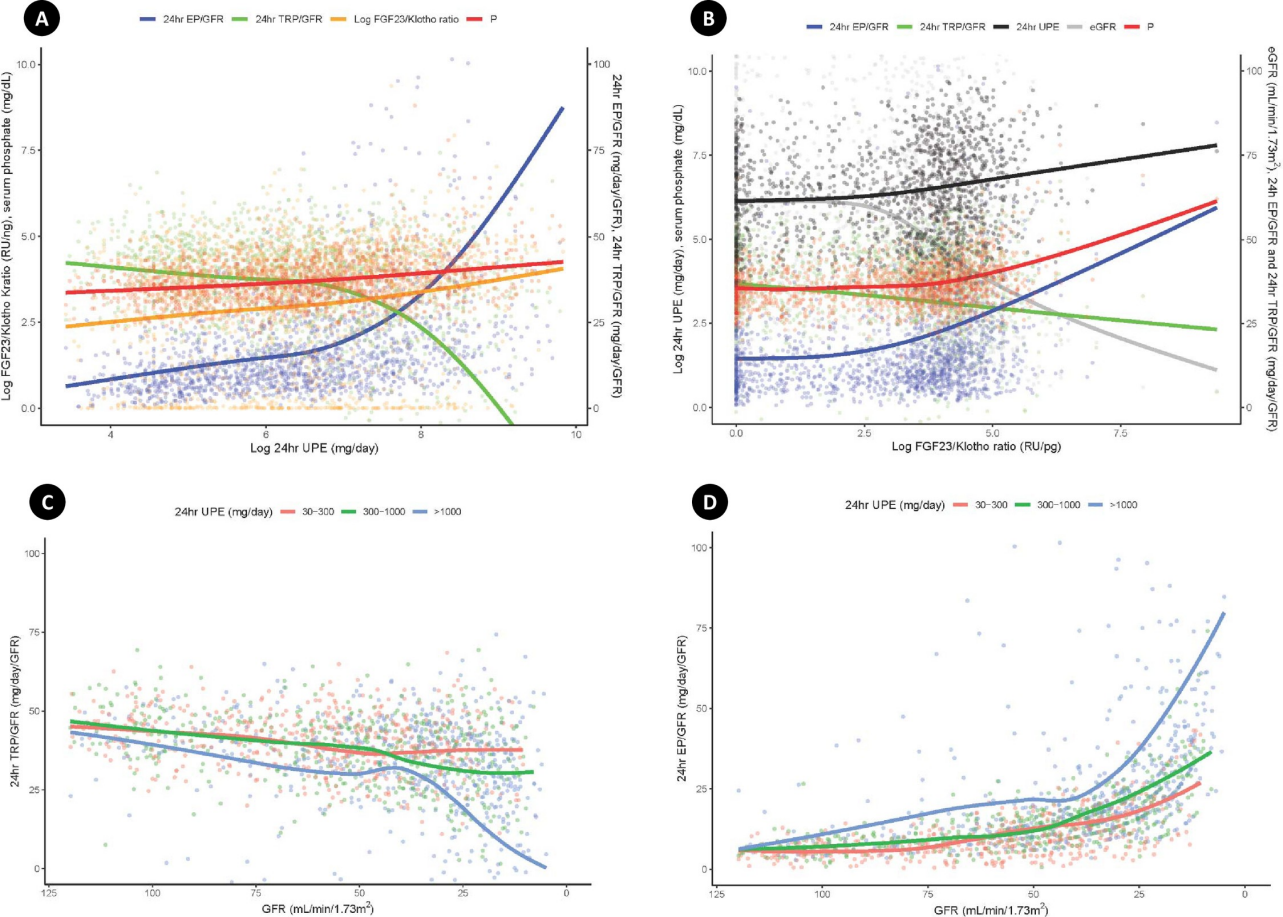
Composite relationships to 24-h UPE, the FGF23/klotho ratio, and the GFR. (A) The composite figure shows that the FGF23/klotho ratio, 24-h EP/GFR, and serum phosphate level increase steadily with the 24-h UPE, while the 24-h TRP/GFR decreases steadily. (B) The 24-h UPE, 24-h EP/GFR, and serum phosphate level increase steadily with the FGF23/klotho ratio as the GFR and 24-h TRP/GFR decrease steadily. (C) As the GFR decreases, the 24-h TRP/GFR decreases gradually. This effect is seen earlier in patients with severe proteinuria. (D) As the GFR decreases, the 24-h EP/GFR increases significantly. This increase occurs more rapidly in patients with severe proteinuria.

Serum phosphate levels were consistently higher among patients with more severe proteinuria, reflecting CKD progression (Fig 1A). A similar trend was seen for the serum FGF23/klotho ratio, which increased with the degree of proteinuria as CKD progressed (Fig 1B). A significant decrease in the 24-h TRP/GFR and increase in the 24-h EP/GFR were also observed in association with the proteinuria increase as CKD progressed (Fig 1C and 1D). We attempted to determine the complexity of the relationship of changes in the FGF23/klotho ratio, 24-h TRP/GFR, 24-h EP/GFR, and serum phosphate level to changes in proteinuria and renal function. The FGF23/klotho ratio, 24-h EP/GFR, and serum phosphate level increased steadily over time, while the 24-h TRP/GFR decreased steadily (Fig 2A and 2B). As the GFR decreased, the 24-h TRP/GFR decreased gradually and the 24-h EP/GFR increased significantly. These effects were seen earlier and occurred more rapidly in association with the severity of proteinuria (Fig 2C and 2D). In contrast, no significant change in the 24-h EP/GFR was observed in association with underlying diseases, including DM, HTN, and glomerulonephritis (S2 Fig in S1 Data).

### Mediation analysis results

We first performed simple mediation analyses with the FGF23 and klotho levels, FGF23/klotho ratio, FEP, and 24-h EP/GFR. Table 3 shows all path coefficients obtained in the simple mediator model. The FGF23 level (effect size, 0.006; 95% CI, 0.001 to 0.014) and the 24-h EP/GFR (effect size, 0.023; 95% CI, 0.008 to 0.038) partially mediated the effect of proteinuria on the serum phosphate level, whereas the klotho level (effect size, 0.003; 95% CI, –0.001 to 0.008) and FEP (effect size, –0.001; 95% CI, –0.003 to 0.002) showed no mediation effect (Table 3). In combinatorial analyses, the FGF23/klotho ratio (effect size, 0.007; 95% CI, 0.001 to 0.015; Table 3) exhibited the strongest mediation effect, which was greater than that of the FGF23 level alone.

**Table 3:** Results of simple mediation analyses.

Proteinuria (exposure)	Serum phosphate (outcome)	
Mediators	Effect ± SE	95% CI
**FGF23**		
***c***	0.044 ± 0.021	0.002–0.086
***c'***	0.038 ± 0.021	- 0.004–0.079
***ab***	0.006 ± 0.003	0.001–0.014
**Klotho**		
***c***	0.044 ± 0.021	0.002–0.086
***c'***	0.041 ± 0.021	- 0.001–0.083
***ab***	0.003 ± 0.002	- 0.001–0.008
**FGF23/Klotho**		
***c***	0.044 ± 0.021	0.002–0.086
***c'***	0.037 ± 0.021	- 0.004–0.079
***ab***	0.007 ± 0.004	0.001–0.015
**FEP**		
***c***	0.044 ± 0.021	0.004–0.087
***c'***	0.045 ± 0.021	0.004–0.088
***ab***	- 0.001 ± 0.001	- 0.003–0.002
**24hr EP/GFR**		
***c***	0.044 ± 0.021	0.002–0.086
***c'***	0.021 ± 0.022	- 0.023–0.065
***ab***	0.023 ± 0.008	0.008–0.038

Covariates; age, sex, smoking, BMI, DM, HTN, CCI, RAAS blockers, PTH, DPI, hsCRP and eGFR.

Mediation effects are calculated by multiplying coefficients of path *a* and path *b* and tested for significance using a bootstrapping (10,000 times) approach.

Estimate coefficient (Coeff.) and standard Error (SE) are expressed according to increasing 24hr UPE categories (<30, 30–300, 300–1000, ≥1000 mg/day).

DPI, hsCRP, FGF23, Klotho and 24hr EP/GFR have been natural log-transformed due to skewed distribution.

**Fig 3:**
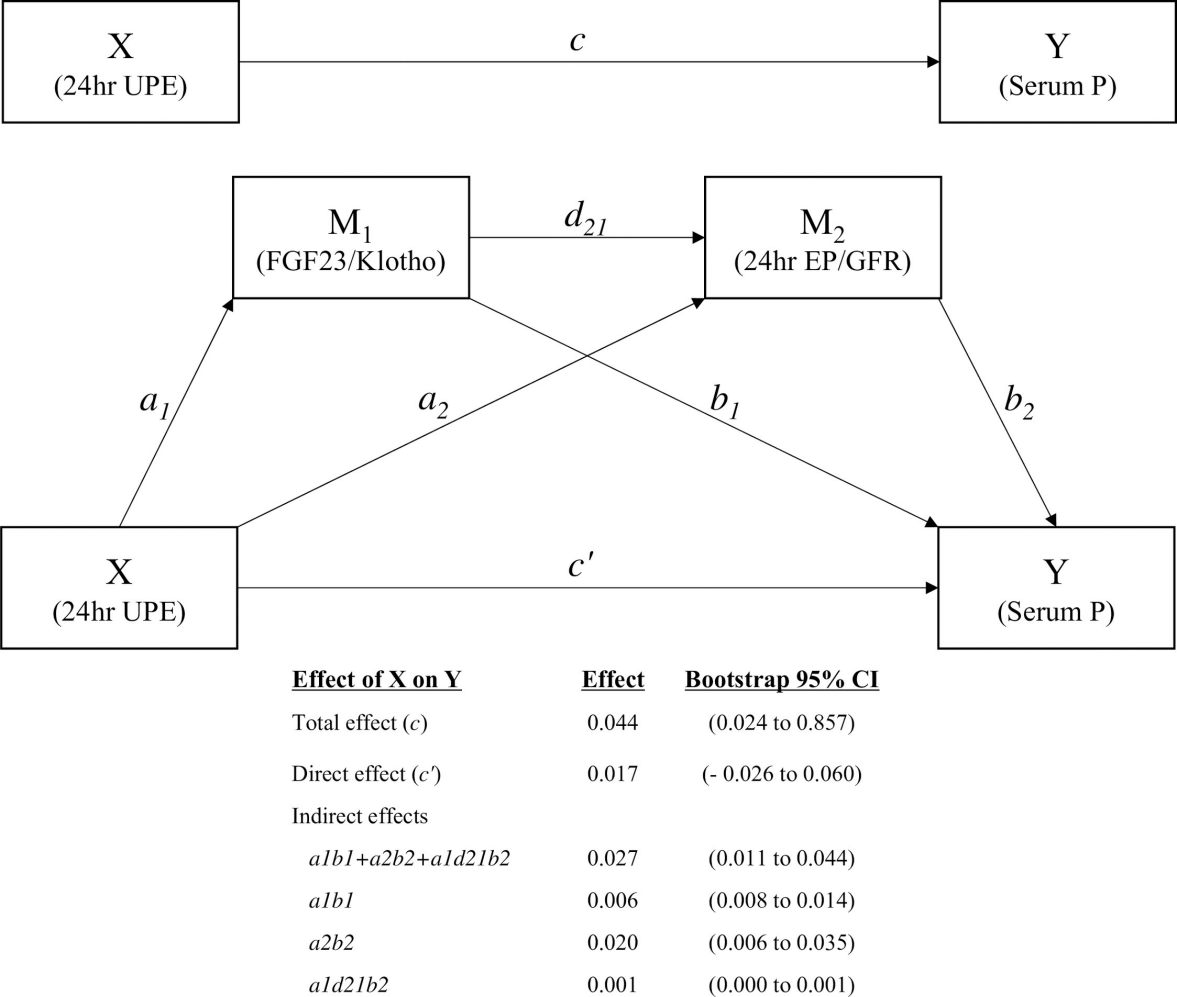
Mediation analyses of the effect of 24-h UPE on the serum phosphate level. Path models and mediation analyses describing mediation of the effect of 24-h UPE on the serum phosphate level by the serum FGF23/klotho ratio and 24-h EP/GFR. The models were adjusted for age, sex, smoking, BMI, DM, CCI, HTN, RAAS blocker use, PTH level, DPI, hsCRP level, and eGFR.

We performed a second round of mediation analyses using the FGF23/klotho ratio and 24-h EP/GFR as mediators in a serial two-mediator model. Path models describing mediation of the relationship between 24-h UPE and the serum phosphate level by the serum FGF23/klotho ratio and 24-h EP/GFR are depicted in Fig 3. Regression Coeffs., SEs, and model summary information for the presumed influence of the two serial-mediator model are presented in Table 4.

**Table 4:** Regression coefficients, standard errors, and model summary information for the presumed media influence of the serial multiple mediator model.

	Consequent											
		M1 (FGF23/Klotho)				M2 (24hr EP/GFR)				Y (serum P)		
Antecedent		Coeff.	*SE*	*P*		Coeff.	*SE*	*P*		Coeff.	*SE*	*P*
**X (24hr UPE)**	*a*_*1*_	0.154	0.064	< 0.001	*a*_*2*_	0.224	0.021	< 0.001	*c’*	0.017	0.022	0.444
*M*_*1*_ (FGF23/Klotho)		―	―	―	*d*_*21*_	0.029	0.010	< 0.001	*b*_*1*_	0.042	0.010	< 0.001
*M*_*2*_ (24hr EP/GFR)		―	―	―		―	―	―	*b*_*2*_	0.091	0.031	0.003
Constant	*i*_*M1*_	3.618	0.621	< 0.001	*i*_*M2*_	2.226	0.206	< 0.001	*i*_*Y*_	3.905	0.218	< 0.001
		*R*^*2*^ = 0.126				*R*^*2*^ = 0.570				*R*^*2*^ = 0.265		
		*F*(13, 1079) = 11.943, *P* < 0.001				*F*(14, 1078) = 102.123, *P* < 0.001				*F*(15, 1077) = 25.900, *P* < 0.001		

Covariates; age, sex, smoking, BMI, DM, HTN, CCI, RAAS blockers, PTH, DPI, hsCRP and eGFR.

Estimate coefficient (Coeff.) and standard Error (SE) are expressed according to increasing 24hr UPE categories (<30, 30–300, 300–1000, ≥1000 mg/day).

DPI, hsCRP, FGF23, Klotho and 24hr EP/GFR have been natural log-transformed due to skewed distribution.

All indirect pathways exhibited significant relationships between variables; however, the direct effect of the association between 24-h UPE and the serum phosphate level was significantly attenuated, and the model was no longer significant after inclusion of the serum FGF23/klotho ratio and 24-h EP/GFR (direct effect size, 0.017; 95% CI, –0.026 to 0.060; Fig 3).

**Fig 4:**
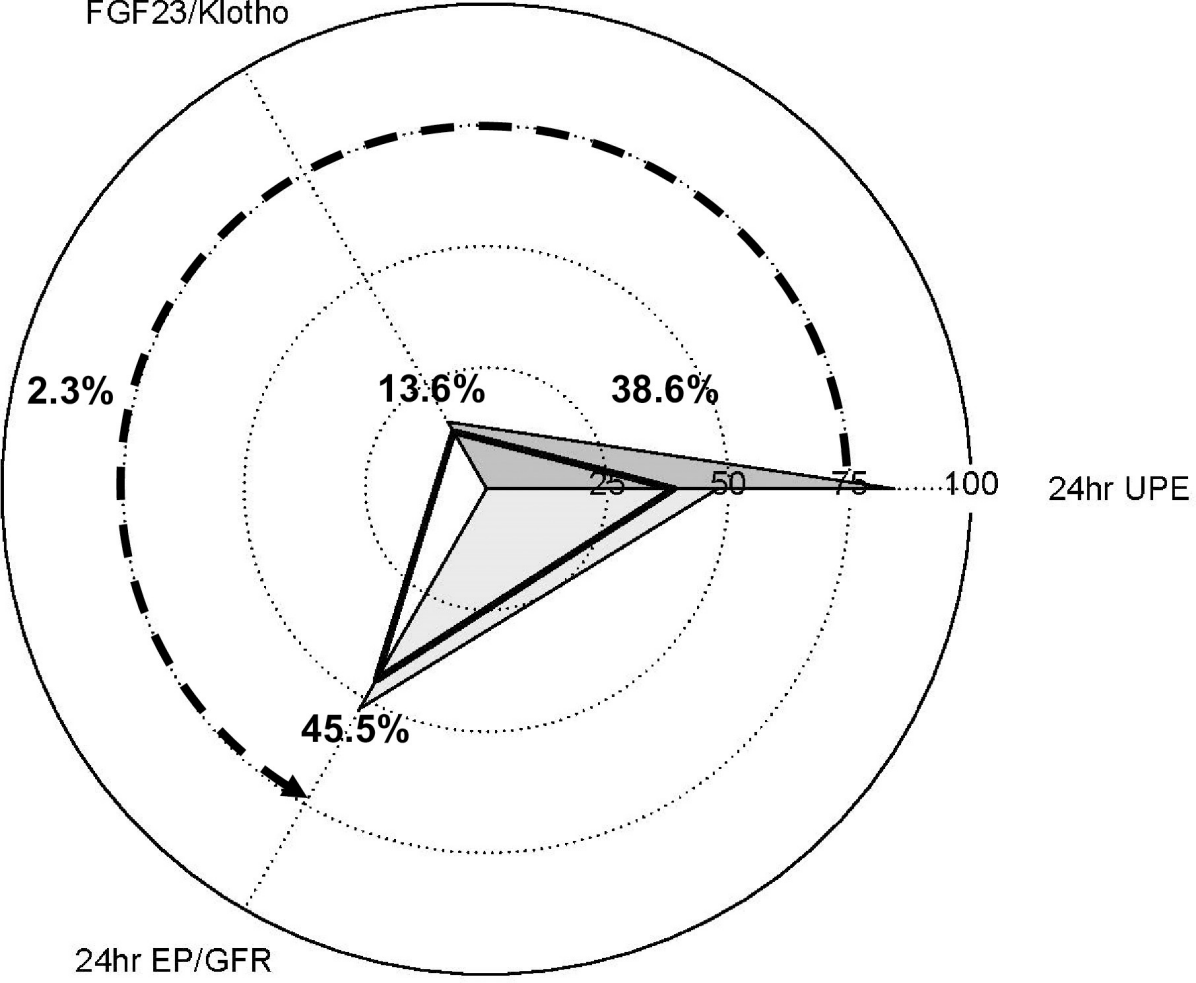
Patterns of direct and mediating effects of 24-h UPE on the serum phosphate level. The radar plot depicts the relative direct effect of 24-h UPE and mediating effects of the FGF23/klotho ratio and 24-h EP/GFR. The relative direct effect of 24-h UPE was calculated as the direct effect divided by the total effect. The relative mediating effects were calculated as the mediating effects divided by the total effect. The solid triangular regions represent the relative direct effect of 24-h UPE and mediating effects from the simple mediation analyses, with the FGF23/klotho ratio and 24-h EP/GFR serving as single mediators. The open triangular regions represent the relative direct effect of 24-h UPE and mediating effects from the serial multiple-mediator model, with the FGF23/klotho ratio and 24-h EP/GFR serving as two sequential mediators.

In Fig 4, we visualize the patterns of relative direct and mediation effects between 24-h UPE and the serum phosphate level. In the serial multiple-mediator model, the mediation effects of the FGF23/klotho ratio and 24-h EP/GFR were 13.6% and 45.5%, respectively, of the total effect, with an additional 2.3% contributed to the effect when these variables were used sequentially. The relative indirect effect of 24-h UPE on the serum phosphate level was 38.6% of the total in the serial two-mediator model.

### Sensitivity analysis results

To further validate our findings, we performed two rounds of sensitivity analysis, excluding patients with GFRs < 15 mL/min/1.73 m^2^ (*n* = 1599; S1 Table in S1 Data) and those with 24-h UPEs ≥ 3500 mg/day (*n* = 1520; S2 Table in S1 Data). All indirect pathways exhibited significant relationships among variables, although the direct effects of the association between 24-h UPE and the serum phosphate level were attenuated significantly after inclusion of the serum FGF23/klotho ratio and 24-h EP/GFR (S3 and S4 Figs).

## Discussion

This study presents noteworthy findings which may be useful in the pre-dialysis diagnosis of CKD. First, the degree of proteinuria correlated strongly with the serum FGF23/klotho ratio and 24-h EP/GFR, and these variables were related to the serum phosphate level. Second, mediation analysis including the FGF23/klotho ratio and 24-h EP/GFR showed that the degree of proteinuria and serum phosphate level were attenuated strongly when these factors were added to the analysis. Together, these observations suggest a causal relationship between proteinuria and the serum phosphate level, such that proteinuria affects the FGF23 and klotho levels, resulting in an increased phosphate burden per unit nephron, irrespective of the GFR, and elevation of the serum phosphate level.

The systemic regulation of phosphate homeostasis is controlled by a complex set of interactions involving the small intestine, bone, parathyroid gland, and kidney [24, 25]. In addition, renal phosphate regulation involves FGF23 and its co-receptor klotho, which in turn affects the NaPi-2a concentration in the renal proximal tubule [26, 27]. Thus, phosphate excretion increases as the GFR falls if this process is not accompanied by an influx of phosphate into the systemic circulation, even when the FGF23 expression level is high [24, 25, 28–31]. In previous studies, serum levels of FGF23 were elevated in pediatric patients with nephrotic-range proteinuria and in patients with IgA nephropathy and proteinuria [8, 9]. We also demonstrated that the 24-h UPE and FGF23 level tended to increase and the klotho level tended to decrease in patients with higher serum phosphate levels.

When the GFR and proteinuria were considered together with the serum phosphate level, the 24-h TRP/GFR was found to decrease and the 24-h EP/GFR was found to increase in response to increasing proteinuria, even in patients with similar GFRs, resulting in increased phosphate burden per unit nephron. Thus, renal activity levels can be assumed to be higher, facilitating increased phosphate excretion, in patients with higher proteinuria relative to those with similar renal function but less proteinuria. The 24-h UPE to creatinine clearance ratio was shown to be associated with more rapid CKD progression, suggesting a harmful effect of an increased phosphate burden per unit nephron prior to an increase in the serum phosphate level [32].

Previous studies have revealed a link between proteinuria and renal klotho expression [33–36]. In patients receiving interventions to reduce proteinuria, renal klotho expression and the circulating α-klotho level were elevated, indicating that the expression and secretion of klotho resulted in a decrease in proteinuria [37, 38]. *In vitro* studies have shown that albumin directly reduces klotho expression in cultured tubular cells [33], and the FGF23 level has been associated with proteinuria in patients with CKD [9, 39]. Other researchers have suggested that a reduction in proteinuria leads to a concomitant decrease in FGF23 expression, which together help to protect kidney function [40]. Although phosphate has been shown to interfere with antiproteinuric responses in patients with very-low-protein diets [17], the use of a high-phosphate food additive did not induce albuminuria [18]. Thus, when explaining the relationship between proteinuria and the serum phosphate level, the assertion that that serum phosphate elevation alone increases urinary excretion, induces tubular damage, and eventually causes proteinuria is not reasonable. Instead, proteinuria-induced stimulation can be assumed to affect the intestinal uptake and transcellular redistribution of phosphate, leading to an increase in systemic influx, although further studies are necessary to confirm this hypothesis.

In this study, we used a mediation model to better understand the mechanism underlying the observed relationship between proteinuria and the serum phosphate level. Our results support the influence of proteinuria on FGF23/klotho expression, indicating a direct causal relationship that ultimately affects the phosphate balance via its effects on tubular phosphate regulation. We also examined the association between proteinuria and the serum phosphate level after excluding patients with GFRs < 15 mL/min/1.73m^2^ (*n* = 1599; S1 Table in S1 Data) or 24-h UPEs ≥ 3500 mg/day. This analysis showed that the FGF23 and klotho levels and their tubular phosphate regulation are indispensable factors in the regulation of phosphate balance, relative to other variables that might affect the relationship between proteinuria and the serum phosphate level. In addition, consideration of the FGF23/klotho ratio, rather than the FGF23 or klotho level alone, may be more suitable for the prediction of renal phosphate regulation, as these factors account for the increment of phosphate burden per unit nephron in patients with CKD and proteinuria.

To account for proteinuria-induced FGF23 resistance at the molecular level, a megalin/cubulin system is required for endocytosis and lysosomal degradation of proximal tubule NPT2a. This system is also used for the reabsorption of albumin. Hence, we could hypothesize that the increased severity of proteinuria in patients with CKDF and increased FGF23/klotho ratios results in decreased phosphate excretion due to decreased endocytosis and degradation of NaPi via the megalin/cubulin system [41–43]. However, the 24-h TRP/GFR was inversely proportional to the degree of proteinuria in this study, which is not consistent with this hypothesis.

Recently, diurnal variation in the regulation of serum phosphate was reported [44, 45]. In addition to dietary intake and renal phosphate regulation by FGF23 and PTH, the role of the nicotinamide phosphoribosyltransferase (Nampt)/nicotinamide adenine dinucleotide (NAD^+^) system on phosphate transfer in the liver, kidney, intestine, and soft tissues has been emphasized [44, 46]. In this study, blood samples were obtained after fasting, preventing us from considering diurnal variation in the regulation of serum phosphate. Thus, future studies should consider the effects of serum phosphate levels at the times of measurement. In addition, the examination of variations in phosphate distribution associated with the Nampt/NAD^+^ system in patients with CKD and proteinuria is important.

In our study, FGF23 levels were measured using an ELISA kit that quantifies intact FGF23 and cFGF23. Although it is physiologically correct that intact FGF23 represents phosphaturic activity, its use has been reported to be unfavorable due to diurnal and intraindividual variations in the phosphate level [47]. In addition, only cFGF23 is detected in patients with normal renal function, and it has been reported to correlate strongly with the serum phosphate level. In our study, serum phosphate levels were measured based on fasting blood samples and 24-h UPE, and patients with near-normal renal function were included. For these reasons, we determined that cFGF23 measurement was more reasonable. However, future studies should examine interactions between FGF23 measurement methods and variables involved in serum phosphate regulation.

Strengths of this study include the use of 24-h urine collections from a large cohort, the concurrent availability of multiple markers of mineral metabolism, and adjustment for confounding factors that may affect serum phosphate levels. However, our study has several limitations. First, due to the observational nature of the study, we could not exclude the possible effects of residual confounding factors, such as the ability to regulate phosphate in the digestive system or a change in this ability depending on the degree of proteinuria. Second, we did not control for variables that could affect the independent and dependent variables, such as medication use or dietary control of protein and phosphate. Although we performed mediation and sensitivity analyses to speculate on the association between proteinuria and the serum phosphate level, to increase the robustness of our findings, a more precisely designed controlled study is warranted to verify and expand on the potential mechanistic link between these factors.

In summary, patients with higher serum phosphate levels were likely to have more severe proteinuria and higher FGF23 levels, and lower klotho levels. In mediation analyses, the association between proteinuria and the serum phosphate level was found to be mediated by the FGF23/klotho ratio and 24-h EP/GFR. Together, these data demonstrate a causal relationship between proteinuria and the serum phosphate level, suggesting the use of the FGF23/klotho ratio and 24-h EP/GFR as mediation factors to provide insight into the mechanism potential underlying this relationship. We believe that these results provide valuable insight into the relationship between proteinuria and the serum phosphate level in patients with CKD.

## Supporting information

S1 Data (PDF)

## References

[1] Stehouwer CD, Smulders YM. Microalbuminuria and risk for cardiovascular disease: Analysis of potential mechanisms. J Am Soc Nephrol. 2006;17(8):2106–11. Epub 2006/07/11. 10.1681/ASN.2005121288 . 16825333

[2] Weir MR. Microalbuminuria and cardiovascular disease. Clin J Am Soc Nephrol. 2007;2(3):581–90. Epub 2007/08/19. 10.2215/CJN.03190906 . 17699466

[3] Mathew S, Tustison KS, Sugatani T, Chaudhary LR, Rifas L, Hruska KA. The mechanism of phosphorus as a cardiovascular risk factor in CKD. J Am Soc Nephrol. 2008;19(6):1092–105. Epub 2008/04/18. 10.1681/ASN.2007070760 18417722 PMC2396927

[4] Scialla JJ, Wolf M. Roles of phosphate and fibroblast growth factor 23 in cardiovascular disease. Nat Rev Nephrol. 2014;10(5):268–78. Epub 2014/04/02. 10.1038/nrneph.2014.49 . 24686452

[5] Tenten V, Menzel S, Kunter U, Sicking EM, van Roeyen CR, Sanden SK, et al Albumin is recycled from the primary urine by tubular transcytosis. J Am Soc Nephrol. 2013;24(12):1966–80. Epub 2013/08/24. 10.1681/ASN.2013010018 23970123 PMC3839546

[6] Gekle M. Renal Proximal Tubular Albumin Reabsorption: Daily Prevention of Albuminuria. News Physiol Sci. 1998;13:5–11. Epub 2001/06/08. 10.1152/physiologyonline.1998.13.1.5 . 11390751

[7] Gattineni J, Bates C, Twombley K, Dwarakanath V, Robinson ML, Goetz R, et al FGF23 decreases renal NaPi-2a and NaPi-2c expression and induces hypophosphatemia in vivo predominantly via FGF receptor 1. Am J Physiol Renal Physiol. 2009;297(2):F282–91. Epub 2009/06/12. 10.1152/ajprenal.90742.2008 19515808 PMC2724258

[8] Feinstein S, Becker-Cohen R, Rinat C, Frishberg Y. Hyperphosphatemia is prevalent among children with nephrotic syndrome and normal renal function. Pediatr Nephrol. 2006;21(10):1406–12. Epub 2006/08/10. 10.1007/s00467-006-0195-2 . 16897004

[9] Lundberg S, Qureshi AR, Olivecrona S, Gunnarsson I, Jacobson SH, Larsson TE. FGF23, albuminuria, and disease progression in patients with chronic IgA nephropathy. Clin J Am Soc Nephrol. 2012;7(5):727–34. Epub 2012/03/03. 10.2215/CJN.10331011 22383747 PMC3338280

[10] Tan SJ, Smith ER, Holt SG, Hewitson TD, Toussaint ND. Soluble klotho may be a marker of phosphate reabsorption. Clin Kidney J. 2017;10(3):397–404. Epub 2017/06/16. 10.1093/ckj/sfw146 28616218 PMC5466110

[11] Dermaku-Sopjani M, Sopjani M, Saxena A, Shojaiefard M, Bogatikov E, Alesutan I, et al Downregulation of NaPi-IIa and NaPi-IIb Na-coupled phosphate transporters by coexpression of Klotho. Cell Physiol Biochem. 2011;28(2):251–8. Epub 2011/08/26. 10.1159/000331737 . 21865732

[12] Phelps KR. Tradeoff-in-the-Nephron: A Theory to Explain the Primacy of Phosphate in the Pathogenesis of Secondary Hyperparathyroidism. Nutrients. 2017;9(5). Epub 2017/04/27. 10.3390/nu9050427 28445401 PMC5452157

[13] Phelps KR, Lieberman RL. Fractional excretion and reabsorption in chronic kidney disease. Clin Nephrol. 2012;77(6):484–90. Epub 2012/05/19. 10.5414/cn107298 . 22595391

[14] Phelps KR, Mason DL. Parameters of phosphorus homeostasis at normal and reduced GFR: theoretical considerations. Clin Nephrol. 2015;83(3):167–76. Epub 2015/02/17. 10.5414/cn108367 . 25685872

[15] Phelps KR, Mason DL, Stote KS. Parameters of phosphorus homeostasis at normal and reduced GFR: empiric observations. Clin Nephrol. 2015;83(4):208–17. Epub 2015/02/25. 10.5414/CN108380 . 25707455

[16] Kim H, Park J, Nam KH, Jhee JH, Yun HR, Park JT, et al The effect of interactions between proteinuria, activity of fibroblast growth factor 23 and serum phosphate on renal progression in patients with chronic kidney disease: a result from the KoreaN cohort study for Outcome in patients With Chronic Kidney Disease study. Nephrol Dial Transplant. 2019 Epub 2019/01/08. 10.1093/ndt/gfy403 . 30615179

[17] Di Iorio BR, Bellizzi V, Bellasi A, Torraca S, D'Arrigo G, Tripepi G, et al Phosphate attenuates the anti-proteinuric effect of very low-protein diet in CKD patients. Nephrol Dial Transplant. 2013;28(3):632–40. Epub 2012/11/21. 10.1093/ndt/gfs477 . 23166309

[18] Chang AR, Miller ER 3rd, Anderson CA, Juraschek SP, Moser M, White K, et al Phosphorus Additives and Albuminuria in Early Stages of CKD: A Randomized Controlled Trial. Am J Kidney Dis. 2017;69(2):200–9. Epub 2016/11/21. 10.1053/j.ajkd.2016.08.029 27865566 PMC5263092

[19] Oh KH, Park SK, Park HC, Chin HJ, Chae DW, Choi KH, et al KNOW-CKD (KoreaN cohort study for Outcome in patients With Chronic Kidney Disease): design and methods. BMC Nephrol. 2014;15:80 Epub 2014/06/03. 10.1186/1471-2369-15-80 24884708 PMC4050398

[20] Levey AS, Stevens LA, Schmid CH, Zhang YL, Castro AF 3rd, Feldman HI, et al A new equation to estimate glomerular filtration rate. Ann Intern Med. 2009;150(9):604–12. Epub 2009/05/06. 10.7326/0003-4819-150-9-200905050-00006 19414839 PMC2763564

[21] Maroni BJ, Steinman TI, Mitch WE. A method for estimating nitrogen intake of patients with chronic renal failure. Kidney Int. 1985;27(1):58–65. Epub 1985/01/01. 10.1038/ki.1985.10 . 3981873

[22] Whitlock G, Lewington S, Sherliker P, Clarke R, Emberson J, Halsey J, et al Body-mass index and cause-specific mortality in 900 000 adults: collaborative analyses of 57 prospective studies. Lancet. 2009;373(9669):1083–96. Epub 2009/03/21. 10.1016/S0140-6736(09)60318-4 19299006 PMC2662372

[23] Hayes AF, Little TD. Introduction to mediation, moderation, and conditional process analysis a regression-based approach. New York, N.Y: The Guilford Press; 2018.

[24] Razzaque MS. The FGF23-Klotho axis: endocrine regulation of phosphate homeostasis. Nat Rev Endocrinol. 2009;5(11):611–9. Epub 2009/10/22. 10.1038/nrendo.2009.196 19844248 PMC3107967

[25] Martin A, David V, Quarles LD. Regulation and function of the FGF23/klotho endocrine pathways. Physiological reviews. 2012;92(1):131–55. Epub 2012/02/03. 10.1152/physrev.00002.2011 22298654 PMC3306265

[26] Ide N, Olauson H, Sato T, Densmore MJ, Wang H, Hanai JI, et al In vivo evidence for a limited role of proximal tubular Klotho in renal phosphate handling. Kidney Int. 2016;90(2):348–62. Epub 2016/06/14. 10.1016/j.kint.2016.04.009 . 27292223

[27] Ide N, Ye R, Courbebaisse M, Olauson H, Densmore MJ, Larsson T, et al In vivo evidence for an interplay of FGF23/Klotho/PTH axis on the phosphate handling in renal proximal tubules. Am J Physiol Renal Physiol. 2018;315(5):F1261–f70. Epub 2018/07/12. 10.1152/ajprenal.00650.2017 29993278 PMC6293295

[28] Kuro OM. The Klotho proteins in health and disease. Nat Rev Nephrol. 2019;15(1):27–44. Epub 2018/11/21. 10.1038/s41581-018-0078-3 . 30455427

[29] Kuro o M. Overview of the FGF23-Klotho axis. Pediatr Nephrol. 2010;25(4):583–90. Epub 2009/07/25. 10.1007/s00467-009-1260-4 . 19626341

[30] Kuro o M. Klotho, phosphate and FGF-23 in ageing and disturbed mineral metabolism. Nat Rev Nephrol. 2013;9(11):650–60. Epub 2013/06/19. 10.1038/nrneph.2013.111 . 23774819

[31] Razzaque MS, Lanske B. The emerging role of the fibroblast growth factor-23-klotho axis in renal regulation of phosphate homeostasis. J Endocrinol. 2007;194(1):1–10. Epub 2007/06/27. 10.1677/JOE-07-0095 17592015 PMC2900827

[32] Kawasaki T, Maeda Y, Matsuki H, Matsumoto Y, Akazawa M, Kuyama T. Urinary phosphorus excretion per creatinine clearance as a prognostic marker for progression of chronic kidney disease: a retrospective cohort study. BMC Nephrol. 2015;16:116 Epub 2015/07/29. 10.1186/s12882-015-0118-1 26215643 PMC4517498

[33] Fernandez-Fernandez B, Izquierdo MC, Valino-Rivas L, Nastou D, Sanz AB, Ortiz A, et al Albumin downregulates Klotho in tubular cells. Nephrol Dial Transplant. 2018;33(10):1712–22. Epub 2018/02/10. 10.1093/ndt/gfx376 . 29425318

[34] Kim JH, Xie J, Hwang KH, Wu YL, Oliver N, Eom M, et al Klotho May Ameliorate Proteinuria by Targeting TRPC6 Channels in Podocytes. J Am Soc Nephrol. 2017;28(1):140–51. Epub 2016/05/07. 10.1681/ASN.2015080888 27151926 PMC5198269

[35] Ozeki M, Fujita S, Kizawa S, Morita H, Sohmiya K, Hoshiga M, et al Association of serum levels of FGF23 and alpha-Klotho with glomerular filtration rate and proteinuria among cardiac patients. BMC Nephrol. 2014;15:147 Epub 2014/09/10. 10.1186/1471-2369-15-147 25200959 PMC4167507

[36] Silva AP, Mendes F, Pereira L, Fragoso A, Goncalves RB, Santos N, et al Klotho levels: association with insulin resistance and albumin-to-creatinine ratio in type 2 diabetic patients. Int Urol Nephrol. 2017;49(10):1809–14. Epub 2017/07/06. 10.1007/s11255-017-1646-3 . 28677090

[37] Lim SC, Liu JJ, Subramaniam T, Sum CF. Elevated circulating alpha-klotho by angiotensin II receptor blocker losartan is associated with reduction of albuminuria in type 2 diabetic patients. J Renin Angiotensin Aldosterone Syst. 2014;15(4):487–90. Epub 2013/02/06. 10.1177/1470320313475905 . 23380567

[38] Karalliedde J, Maltese G, Hill B, Viberti G, Gnudi L. Effect of renin-angiotensin system blockade on soluble Klotho in patients with type 2 diabetes, systolic hypertension, and albuminuria. Clin J Am Soc Nephrol. 2013;8(11):1899–905. Epub 2013/08/10. 10.2215/CJN.02700313 23929932 PMC3817905

[39] Vervloet MG, van Zuilen AD, Heijboer AC, ter Wee PM, Bots ML, Blankestijn PJ, et al Fibroblast growth factor 23 is associated with proteinuria and smoking in chronic kidney disease: an analysis of the MASTERPLAN cohort. BMC Nephrol. 2012;13:20 Epub 2012/04/26. 10.1186/1471-2369-13-20 22530966 PMC3366907

[40] Yilmaz MI, Sonmez A, Saglam M, Kurt YG, Unal HU, Karaman M, et al Ramipril lowers plasma FGF-23 in patients with diabetic nephropathy. Am J Nephrol. 2014;40(3):208–14. Epub 2014/10/18. 10.1159/000366169 . 25324042

[41] Bachmann S, Schlichting U, Geist B, Mutig K, Petsch T, Bacic D, et al Kidney-specific inactivation of the megalin gene impairs trafficking of renal inorganic sodium phosphate cotransporter (NaPi-IIa). J Am Soc Nephrol. 2004;15(4):892–900. Epub 2004/03/23. 10.1097/01.asn.0000120389.09938.21 . 15034091

[42] de Seigneux S, Courbebaisse M, Rutkowski JM, Wilhelm-Bals A, Metzger M, Khodo SN, et al Proteinuria Increases Plasma Phosphate by Altering Its Tubular Handling. Journal of the American Society of Nephrology: JASN. 2015;26(7):1608–18. Epub 2014/10/29. 10.1681/ASN.2014010104 25349200 PMC4483577

[43] de Seigneux S, Wilhelm-Bals A, Courbebaisse M. On the relationship between proteinuria and plasma phosphate. Swiss medical weekly. 2017;147:w14509 Epub 2017/10/25. 10.4414/smw.2017.14509 . 29063524

[44] Miyagawa A, Tatsumi S, Takahama W, Fujii O, Nagamoto K, Kinoshita E, et al The sodium phosphate cotransporter family and nicotinamide phosphoribosyltransferase contribute to the daily oscillation of plasma inorganic phosphate concentration. Kidney Int. 2018;93(5):1073–85. Epub 2018/02/06. 10.1016/j.kint.2017.11.022 . 29398136

[45] Nomura K, Tatsumi S, Miyagawa A, Shiozaki Y, Sasaki S, Kaneko I, et al Hepatectomy-related hypophosphatemia: a novel phosphaturic factor in the liver-kidney axis. J Am Soc Nephrol. 2014;25(4):761–72. Epub 2013/11/23. 10.1681/ASN.2013060569 24262791 PMC3968501

[46] Ramsey KM, Yoshino J, Brace CS, Abrassart D, Kobayashi Y, Marcheva B, et al Circadian clock feedback cycle through NAMPT-mediated NAD+ biosynthesis. Science. 2009;324(5927):651–4. Epub 2009/03/21. 10.1126/science.1171641 19299583 PMC2738420

[47] Smith ER, Cai MM, McMahon LP, Holt SG. Biological variability of plasma intact and C-terminal FGF23 measurements. J Clin Endocrinol Metab. 2012;97(9):3357–65. Epub 2012/06/13. 10.1210/jc.2012-1811 . 22689697

