# Supplementary material for: Mediation of the relationship between proteinuria and serum phosphate: Insight from the KNOW-CKD study
Source: PLoS One. 2020 Jun 22;15(6):e0235077. doi: 10.1371/journal.pone.0235077 (PMC7307748; doi:10.1371/journal.pone.0235077)
Supplement: S1 Data — (PDF) [file pone.0235077.s001.pdf]

Contents

Supplemental Table S1. 2

Supplemental Table S2. 3

Supplemental Figure S1. 4

Supplemental Figure S2. 5

Supplemental Figure S3. 6

Supplemental Figure S4. 7

(<30, 30–300, 300–1000, ≥1000 mg/day).
DPI, hsCRP, FGF23, Klotho and 24hr EP/GFR have been natural log-transformed due to skewed distribution.

(<30, 30–300, 300–1000, ≥1000 mg/day).
DPI, hsCRP, FGF23, Klotho and 24hr EP/GFR have been natural log-transformed due to skewed distribution.

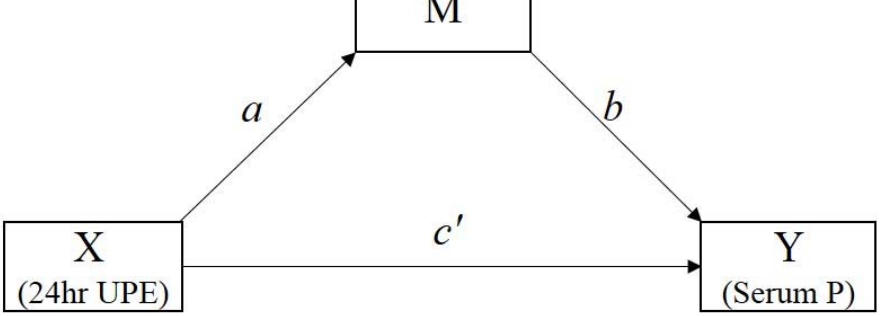

M
a
b
c′
X
(24hr UPE)
Y
(Serum P)

(B)

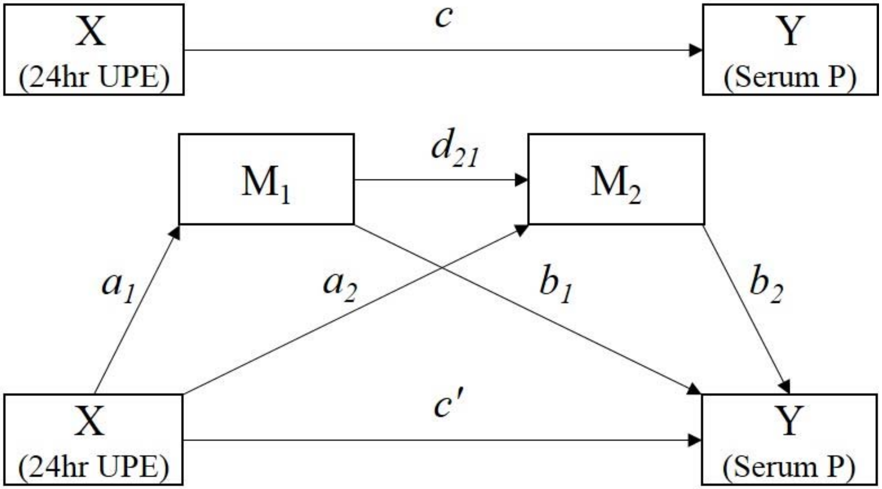

X
(24hr UPE)
c
Y
(Serum P)
M1
d21
M2
a1
a2
b1
b2
X
(24hr UPE)
c′
Y
(Serum P)

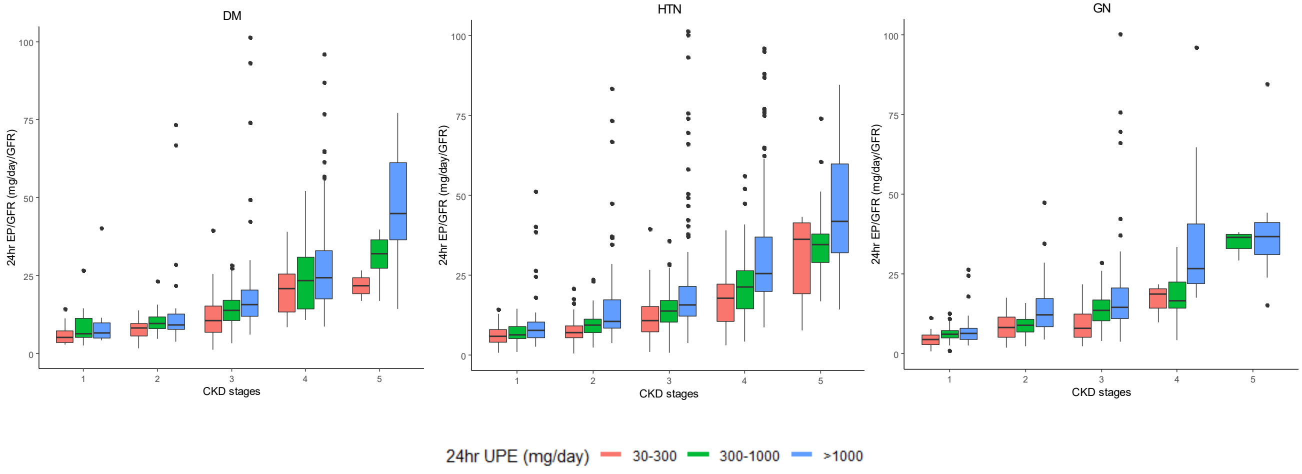
DM
HTN
GN
24hr EP/GFR (mg/day/GFR)
CKD stages
24hr UPE (mg/day)
30-300
300-1000
>1000

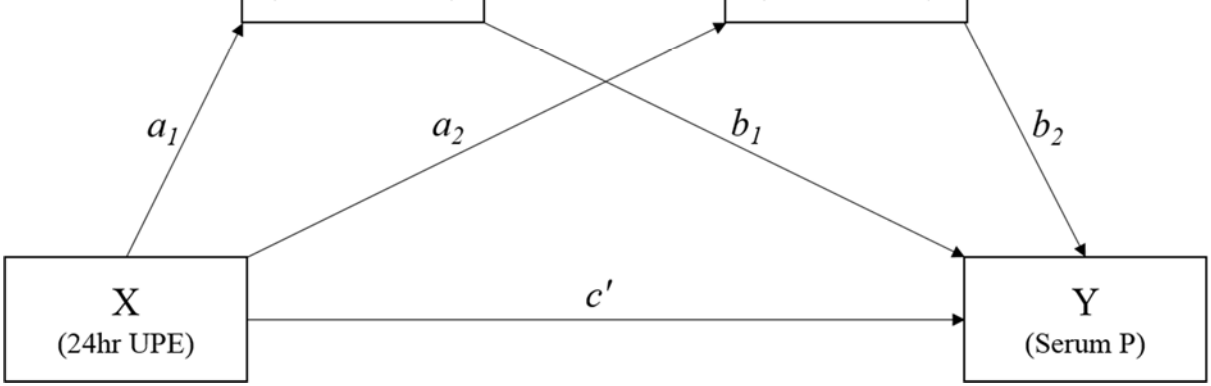

**Effect of X on Y**	**Effect**	**Bootstrap 95% CI**
Total effect (*c*)	0.036	(0.004 to 0.076)
Direct effect (*c*′)	0.020	(- 0.022 to 0.062)
Indirect effects		
a1b1+a2b2+a1d21b2	0.016	(0.003 to 0.030)
a1b1	0.005	(0.001 to 0.010)
a2b2	0.010	(0.000 to 0.024)
a1d21b2	0.001	(0.000 to 0.001)

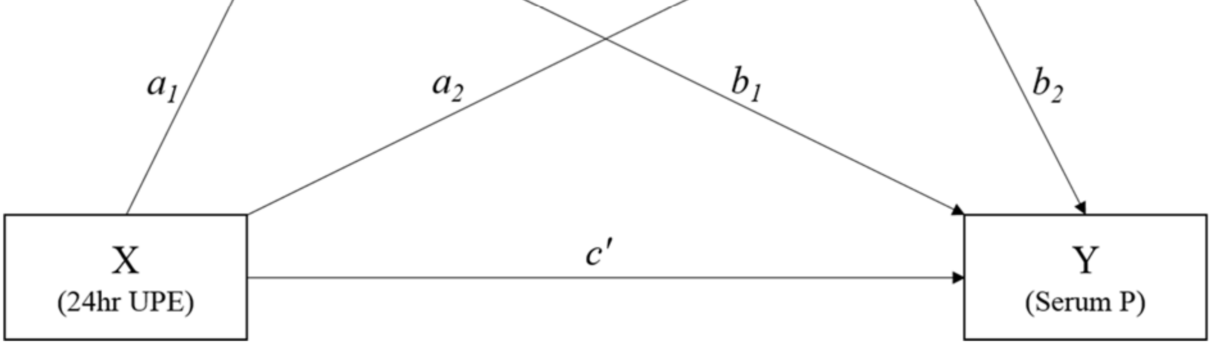

Effect of X on Y	Effect	Bootstrap 95% CI
Total effect (*c*)	0.034	(0.001 to 0.075)
Direct effect (*c'*)	0.019	(- 0.025 to 0.062)
Indirect effects		
a1b1+a2b2+a1d21b2	0.015	(0.000 to 0.028)
a1b1	0.005	(0.000 to 0.012)
a2b2	0.009	(0.000 to 0.021)
a1d21b2	0.001	(0.000 to 0.002)
